# Reliability and Validity of the Korean Version of the Multidimensional Fatigue Inventory (MFI-20): A Multicenter, Cross-Sectional Study

**DOI:** 10.1155/2018/3152142

**Published:** 2018-05-09

**Authors:** Sang-Wook Song, Sung-Goo Kang, Kyung-Soo Kim, Moon-Jong Kim, Kwang-Min Kim, Doo-Yeoun Cho, Young-Sang Kim, Nam-Seok Joo, Kyu-Nam Kim

**Affiliations:** ^1^Department of Family Medicine, St. Vincent's Hospital, College of Medicine, The Catholic University of Korea, Suwon, Republic of Korea; ^2^Department of Family Medicine, CMC Clinical Research Coordinating Center, Seoul St. Mary's Hospital, College of Medicine, The Catholic University of Korea, Seoul, Republic of Korea; ^3^Department of Family Medicine, CHA Bundang Medical Center, CHA Medical University, Seongnam, Republic of Korea; ^4^Department of Family Practice and Community Health, Ajou University School of Medicine, Suwon, Republic of Korea

## Abstract

**Introduction:**

A nonspecific symptom, fatigue accompanies a variety of diseases, including cancer, and can have a grave impact on patients' quality of life. As for multidimensional instruments, one of the most widely used is the Multidimensional Fatigue Inventory (MFI). This study aims to verify the reliability and validity of the MFI Korean (MFI-K) version.

**Materials and Method:**

This study was performed at four university hospitals in the Republic of Korea. Among outpatients visiting the Department of Family Medicine, those complaining of fatigue or visiting a chronic care clinic were enrolled in this study. A total of 595 participants were included, and the mean age was 42.2 years.

**Results:**

The Cronbach's alpha coefficient of the MFI-K was 0.88. The MFI-K had good convergent validity. Most subscales of the MFI-K were significantly correlated with the Visual Analogue Scale (VAS) and Fatigue Severity Scale (FSS). In particular, general and physical fatigue had the greatest correlation with the VAS and FSS. Although the English version of MFI had five subscales, the factor analysis led to four subscales in the Korean version.

**Conclusion:**

This study demonstrated the clinical usefulness of MFI-K instrument, particularly in assessing the degree of fatigue and performing a multidimensional assessment of fatigue.

## 1. Introduction

Fatigue is a largely subjective symptom and is one of the most common symptoms encountered in primary care. A nonspecific symptom, fatigue accompanies a variety of diseases, including cancer, and can have a grave impact on patients' quality of life [1–8]. The medical cause of fatigue remains undiscovered in about 59–64% of adults who claim to have it [1]. Because of this nonspecificity, differential diagnosis and management of fatigue is often difficult. Nevertheless, both patients and physicians alike tend to underestimate fatigue symptoms solely due to their high prevalence.

One study showed that the prevalence of fatigue varies widely in primary practice, from 7 to 45%. One reason for such wide variation might be its ambiguous definition and differences in measurement instruments used [1].

Instruments useful for assessing fatigue can be categorized as unidimensional and multidimensional. The most widely used unidimensional fatigue assessment instruments are the Visual Analogue Scale (VAS) and Fatigue Severity Scale (FSS) developed by Dr. Krupp [9, 10]. The FSS has already been translated into Korean and validated [11]. As for multidimensional instruments, one of the most widely used is the Multidimensional Fatigue Inventory (MFI) [12]. The MFI was developed by E. M. A. Smets, a Dutch doctor. The validities of the English [12], French, and Swedish versions [13, 14] of the scale have been verified. The MFI has five subscales: general fatigue, physical fatigue, reduced activity, reduced motivation, and mental fatigue. Gentile et al. [13] conducted factor analysis at the same time as they conducted a validation study of the French version of MFI. The subscale of the French version of MFI was different from that of the MFI of the original version. The structure of Korean sentences is very different from the structure of English sentences. Korean sentences have the structure of subject + object + verb. Even the meaning of positive and negative is placed at the end of the sentence. Therefore, the subscales of MFI Korean version are very likely to be different from the MFI original version.

Because of cultural differences, translated scales must be appropriately validated to be suitable to the corresponding culture. Although many studies on fatigue in various patient populations, including patients with cancer and COPD, are underway in South Korea, there is practically no multidimensional instrument with verified reliability and validity in Korean. While researchers have translated the MFI into Korean and used it in practice, the reliability and validity of the translated version have not been established. Therefore, this study aims to verify the reliability and validity of the MFI Korean (MFI-K) version and to establish a new factor structure for the Korean outpatient population.

## 2. Materials and Methods

### 2.1. Study Population

The study population comprised adults aged 19 years or older. This study was performed at four university hospitals based in Seoul (*n*=1) and two cities in Gyeonggi Province: Suwon (*n*=2) and Seongnam (*n*=1). Since Seoul is a metropolitan city with a population of more than 10 million, and Seongnam and Suwon are also cities with populations of more than 1 million, the subjects of this study were all urban people. Among outpatients visiting the Department of Family Medicine, those complaining of fatigue or who were visiting a chronic care clinic were enrolled in this study. Individuals without fatigue symptoms who have visited the health improvement center for a routine health examination were established as the control group. On the first page of the questionnaire, participants were asked to choose one of the following responses regarding their level of fatigue over the past week, before continuing with the rest of the questionnaire: (1) rarely, (2) moderate, and (3) very severe.

### 2.2. Translation of MFI-K

The translation was performed after obtaining approval from Dr. Smets, the developer of the MFI. The translation process suggested by Guillemin et al. [15] and Beaton et al. [16] was followed. First, two Korean native translators proficient in English independently translated the English version into Korean. One of these translators was the first author of this study, and the other translator was blinded to the purpose of the questionnaire to enhance the quality of the translation. Next, four specialists discussed and analyzed the differences between the two translations to merge them into a single complete translation. During the back-translation stage, two new back-translators independently translated the completed translation into the source language (English). The back-translators were native English speakers also proficient in Korean. In addition, they were not medical professionals and were blinded to the purpose of the questionnaire. Finally, four specialists discussed and analyzed the back-translated version to complete the final MFI-K. Dr. Smets reviewed the final version and approved the study using MFI-K.

### 2.3. Visual Analogue Scale (VAS)

The VAS is 100 mm long, with the left end indicating “no exhaustion at all” and the right end indicating “complete exhaustion.” We showed participants the VAS and instructed them to put a mark on the VAS line to indicate their level of fatigue. Then we measured the length (mm) from the left end of the line to the mark to indicate their score. A higher score indicated more severe fatigue.

### 2.4. Fatigue Severity Scale (FSS)

To assess fatigue severity, we additionally used the FSS developed by Krupp et al. [10]. The FSS is a 9-item self-report questionnaire. Each item is rated on a seven-point scale, where respondents choose a score between 1 (no impairment at all) and 7 (severe impairment) corresponding to their perceived impairment. The total score is then divided by the total number of items to generate the mean score. This scale was translated into Korean previously and has been found to be clinically useful [11].

### 2.5. Statistical Analysis

All statistical analyses were performed using the PASW Statistics (i.e., SPSS) 18.0 for Windows. Items within the original five factors of the MFI-K were assessed for their internal consistency by using Cronbach's alpha. Principal component analysis was performed to examine the MFI-K's factor structure; factors with eigenvalues of >1 were extracted. The convergent validity of the total cognition score was tested using the Spearman rank-order correlation and Pearson correlation coefficients with the VAS and FSS. Differences between the fatigue groups were tested using the chi-square test and ANOVA.

### 2.6. Ethics Statement

This study was implemented in accordance with ethical and safety guidelines upon the approval of the Institutional Review Board in The Catholic University of Korea, St. Vincent's Hospital (IRB approval number: VC15QIMI0193). We provided adequate explanation of the aim, structure, content, and precautions of the study to all participants, and participants were given sufficient time to make decisions. The participants were then instructed to answer all questionnaire items after reading the instructions of each instrument. They were told to ask any questions they might have during the course of completing the questionnaire to ensure that they accurately understand the instrument items.

## 3. Results

### 3.1. General Characteristics

A total of 595 participants were included, and the mean age was 42.22 ± 12.36 years. A total of 380 (63.99%) participants were women and 215 (36.01%) were men. The study groups did not significantly differ in terms of body mass index, marital status, highest education level, and smoking status. Although statistically nonsignificant, women tended to experience more severe fatigue than did men. Participants who reported severe fatigue were younger and slept significantly less (Table 1).

Fifty-one (8.5%) participants said that the content of the questionnaire was slightly difficult to understand, while 8 participants (1.3%) said that the content of the questionnaire was very difficult to understand. The remaining 538 (90.2%) participants said that the content of the questionnaire was neither difficult nor easy to understand or was easy to understand.

Overall, the MFI-K was well received by the participants. About 99.5% of the participants completed the MFI-K without omissions. Extremely few questionnaires were submitted with missing items (less than 0.5%).

The time taken to complete the MFI-K did not significantly differ among the fatigue groups (4.14 ± 2.95 versus 4.05 ± 3.25 versus 4.36 ± 3.06, *P*=0.557).

### 3.2. Internal Consistency

The Cronbach's alpha coefficient of the MFI-K was 0.88. The internal consistency of each of the five original subscales was as follows: general fatigue (0.84), physical fatigue (0.78), mental fatigue (0.67), reduced activity (0.66), and reduced motivation (0.52).

### 3.3. Structure Validity

The results of the principal component analysis (with a varimax rotation) are shown in Tables 2 and 3. Four factors explained 57.33% of the total variance. Factor 1 explains 31.9% of the total variance and included all of the “general fatigue” items and some “physical fatigue” items. Factor 2 explains 12.4% of the total variance and includes all “mental fatigue” items and “I don't feel like doing anything” items. Factor 3 mostly includes the “reduced activities” items, and most of the items are phrased negatively. Factor 4 included positively worded phrases about motivation. Although the English version had five subscales, the factor analysis led to four subscales in the Korean version.

### 3.4. Convergent Validity

The MFI-K had good convergent validity. Most subscales of the MFI-K were significantly correlated with the VAS. In particular, general fatigue had the greatest correlation with the VAS (*r*=0.533). Reduced activity had the lowest significant correlation with the VAS (*r*=0.084).

Similarly, the general fatigue subscale of the MFI-K had the strongest correlation with the FSS (*r*=0.725). The reduced activity subscale had the lowest correlation with the FSS (*r*=0.162) but the correlation was statistically significant (Table 4). These results were also found for the new factor structure of the MFI-K (Tables 5 and 6).

### 3.5. Differences in MFI-K Scores across Fatigue Groups

The mean MFI-K score significantly varied across the fatigue groups. With the exception of reduced activities, all subgroup scores significantly differed across the groups. The VAS and FSS scores also significantly differed across groups. In all cases, the mean MFI-K score increased with increasing fatigue severity (Table 6).

## 4. Discussion

This study aimed to verify the reliability and validity of the Korean version of the MFI, one of the most widely used multidimensional fatigue scales. It was also the first study to examine a Korean version of the Multidimensional Fatigue Scale.

We tested the reliability of the MFI-K using Cronbach's alpha coefficients. Scales with Cronbach's alpha values of greater than 0.7 are considered reliable [17], and the Cronbach's alpha coefficient of the MFI-K was 0.88, suggesting high reliability. The subscales also showed reasonable internal consistency (>0.65), with the exception of reduced motivation. This was similar to the results presented by Dr. Smets, the developer of the MFI [12]. However, the internal consistency was generally lower than that of the French version of the MFI [13]. Although additional studies are needed to shed light on the causes of the difference between our findings and findings from other countries, it is nevertheless possible that differences in sentence structures are involved. For instance, unlike English and French, the verb is placed at the end of the sentence in Korean, which means that words suggesting a negative or positive tone are placed at the end of the sentence, requiring readers to focus on the end of each sentence in order to accurately understand the whole sentence. As proof of this, factor 3 mostly includes negatively worded phrases (except one item, 6, “I think I do a lot in a day”) and factor 4 included positively worded phrases.

To know this more clearly, further research in Japan and China is needed to confirm whether this is a geographical, cultural, or sentence structural problem. Although Korea, China, and Japan have very close geographical and historical relations, the language structure of Chinese is similar to English, but the language structure of Japanese is similar to Korean. Therefore, if MFI is translated into Chinese and Japanese, and factor analysis is performed, it is possible to know more exactly whether MFI-K differs from MFI of English version because of geographical and cultural differences or differences in language structure.

As anticipated, the general and physical fatigue subscale of the MFI-K had the strongest correlations with the VAS and FSS. This is in line with the findings of Dr. Smets [18] that the general fatigue subscale of the MFI-20 is compatible with the VAS for assessing fatigue in cancer patients, and with the findings of Rupp et al. [4] that the general fatigue and physical fatigue subscales of the MFI-20 had the highest correlations with the VAS in patients with rheumatoid arthritis. The subjects of this study were people who visited family medicine departments for (generally physical) fatigue or visited health promotion centers for routine health checkups. The correlation between general and physical fatigue subscale of the MFI-K and FSS was higher than 0.7 and showed a low correlation with other subscales. These results demonstrate the ability of the MFI-K to differentiate between the degree and the type of fatigue.

As for the structural validity, the MFI-K has only four subscales (Tables 2 and 3), whereas the English version has five. The English version of the MFI includes general fatigue (items 1, 5, 12, and 16), physical fatigue (items 2, 8, 14, and 20), reduced activity (items 3, 6, 10, and 17), reduced motivation (item 4, 9, 15, and 18), and mental fatigue (item 7, 11, 13, and 19) subscales (Table 3). In our study, it was difficult to distinguish general fatigue from physical fatigue, which is similar to the study by Dr. Smets and the study on the Swedish version of the MFI [19]. With the exception of item 2 and item 8, items corresponding to general fatigue and physical fatigue in the English version of MFI consisted of one factor (general and physical fatigue) in MFI-K. In the English version of MFI, item 2 and item 8 are included in the physical fatigue subscale. However, in MFI-K, item 2 (“Physically I feel only able to do a little”) was more relevant to the reduced activities subscale, and item 8 (“Physically I can take on a lot”) was more relevant to the motivation subscale. In the English version of MFI, item 9 and item 18 are included in the reduced motivation subscale. In MFI-K, item 9 (“I dread having to do things”) and item 18 (“I don't feel like doing anything”) were more relevant to the mental fatigue subscale. As mentioned earlier, in MFI-K, factor 3 (reduced activity) mostly included negatively worded phrases (except one item, 6, “I think I do a lot in a day”) and factor 4 (motivation) included positively worded phrases. It is a sentence of the same meaning, but depending on the English or Korean language, it can be conveyed to the person reading the item with a different feeling. In the end, the structure of the MFI-K was generally inconsistent with the English version of the MFI and was closer to the French version.

This study has a few limitations. First, the subjects of this study were all urban people. Rural people were not included in the study. However, as of 2016, 91.8% of the total population of Korea lives in cities [20]. Second, in this study, the employment status of the subjects was not investigated.

This study demonstrated the clinical usefulness of this instrument, particularly in assessing the degree of fatigue and performing a multidimensional assessment of fatigue. The general and physical fatigue subscale was strongly associated with the global fatigue measures (VAS and FSS), and the other subscales had fair relationships with the FSS. These results show that fatigue is a multidimensional concept and various aspects must be considered when dealing with patients with fatigue. Follow-up studies should be conducted on a more diverse study population for whom fatigue assessment using the MFI-K is important, such as cancer patients or families of cancer patients, as well as patients with chronic fatigue syndrome, fibromyalgia, or chronic obstructive pulmonary disease.

## 5. Conclusion

This study demonstrated the clinical usefulness of MFI-K instrument, particularly in assessing the degree of fatigue and performing a multidimensional assessment of fatigue. The MFI-K is the first validated tool to assess various aspects of fatigue in Koreans.

## Figures and Tables

**Table 1:** Baseline characteristics of the study population.

	No or mild fatigue (*N*=76)	Moderate fatigue (*N*=342)	Severe fatigue (*N*=174)	*P* value
Age (years)	45.39 ± 13.68	42.39 ± 12.20	40.49 ± 11.82	0.014
Sex				0.071
Male	36 (16.8)	122 (57.0)	56 (26.2)	
Female	40 (10.6)	222 (28.6)	117 (30.9)	
BMI (kg/m^2^)	24.04 ± 5.36	23.39 ± 10.01	22.79 ± 3.61	0.506
Sleep duration	6.78 ± 1.09	6.43 ± 1.08	6.23 ± 1.31	0.002
Smoking				0.161
Smoker	9 (14.1)	31 (48.4)	25 (37.5)	
Ex-smoker	16 (19.8)	44 (54.3)	21 (25.9)	
Nonsmoker	50 (11.4)	260 (59.5)	127 (29.1)	
Marital condition				0.542
Single	18 (10.7)	98 (58.0)	53 (31.4)	
Married	56 (13.9)	231 (57.3)	116 (28.8)	
Others	4 (19.0)	13 (61.9)	4 (19.0)	
Education				0.362
Under middle school	6 (17.6)	19 (55.9)	9 (26.5)	
High school or college	37 (17.3)	119 (55.6)	58 (27.1)	
Over university	35 (10.1)	205 (59.2)	106 (30.6)	

Others: separation, divorce, or bereavement, data are mean ± SD or *N* (%); *P* values were obtained by the one-way ANOVA or chi-square test.

**Table 2:** Principal component analysis after varimax rotation of MFI Korean version.

Factors	Eigenvalue	% total of variance	Items	Interpretation of Korean dimensions
Factor 1	6.37	31.85	1, 5, 12, 14, 16, 20	General and physical fatigue
Factor 2	2.48	12.41	7, 9, 11, 13, 18, 19	Mental fatigue
Factor 3	1.38	6.92	2, 6, 10, 17	Reduced activity
Factor 4	1.23	6.15	3, 4, 8, 15	Motivation

**Table 3:** Items of the new dimension of the MFI Korean version.

New dimension Korean version	Items contribution
General and physical fatigue	1. 나는 몸 상태가 좋다. (I feel fit.)
	5. 나는 피곤함을 느낀다. (I feel tired.)
	12. 나는 가뿐하다. (I am rested.)
	14. 육체적으로 나는 몸 상태가 나쁘다고 생각한다. (Physically I feel I am in a bad condition.)
	16. 나는 쉽게 피곤해진다. (I tire easily.)
	20. 육체적으로 나는 몸 상태가 아주 좋다고 생각한다. (Physically I feel I am in an excellent condition.)
Mental fatigue	7. 나는 어떤 일을 하는 동안 그 일에 대한 생각을 계속 유지할 수 있다. (When I am doing something, I can keep my thoughts on it.)
	9. 나는 어떤 일을 하는 것이 염려스럽다. (I dread having to do things.)
	11. 나는 집중을 잘 할 수 있다. (I can concentrate well.)
	13. 어떤 일에 집중하기 위해서 많은 노력이 필요하다. (It takes a lot of effort to concentrate on things.)
	18. 나는 어떠한 일도 하고 싶지 않다. (I don't feel like doing anything.)
	19. 생각이 쉽게 산만해진다. (My thoughts easily wander.)
Reduced activities	2. 육체적으로 나는 아주 가벼운 일 밖에 할 수 없다. (Physically I feel only able to do a little.)
	6. 나는 하루 동안에 아주 많은 일을 해낸다고 생각한다. (I think I do a lot in a day.)
	10. 나는 하루 동안에 아주 적은 일을 한다고 생각한다. (I think I do very little in a day.)
	17. 나는 처리한 일이 거의 없다. (I get little done.)
Motivation	3. 나는 매우 활동적이라고 생각한다. (I feel very active.)
	4. 나는 온갖 흥미로운 일들에 빠져들기를 좋아한다. (I feel like doing all sorts of nice things.)
	8. 육체적으로 나는 많은 일을 해낼 수 있다. (Physically I can take on a lot.)
	15. 나는 계획하고 있는 일들이 많다. (I have a lot of plans.)

**Table 4:** Correlation between MFI-K and VAS score and FSS score in English factor structure.

Dimensions: English structure	VAS score	FSS score
General fatigue	0.533^∗∗^	0.725^∗∗^
Physical fatigue	0.378^∗∗^	0.578^∗∗^
Mental fatigue	0.241^∗∗^	0.403^∗∗^
Reduced activity	0.084^∗^	0.162^∗∗^
Reduced motivation	0.276^∗∗^	0.391^∗∗^
MFI-K total score	0.419^∗∗^	0.635^∗∗^

^∗∗^ *P* < 0.01, ^∗^*P* < 0.05; correlation coefficients were obtained by Spearman correlation analysis.

**Table 5:** Correlation between MFI-K and VAS score and FSS score in Korean factor structure.

Dimensions: Korean structure	VAS score	FSS score
General and physical fatigue	0.519^∗∗^	0.718^∗∗^
Mental fatigue	0.333^∗∗^	0.506^∗∗^
Reduced activity	0.087^∗^	0.200^∗∗^
Motivation	0.159^∗∗^	0.248^∗∗^
MFI-K total score	0.419^∗∗^	0.635^∗∗^

^∗∗^ *P* < 0.01, ^∗^*P* < 0.05; correlation coefficients were obtained by Spearman correlation analysis.

**Table 6:** Univariate *F*-test for group differences on subclass of the MFI Korean structure.

	No or mild fatigue	Moderate fatigue	Severe fatigue	*P* value
MFI Korean structure				
General and physical fatigue	14.37 ± 4.73	20.67 ± 4.30	26.83 ± 3.94	<0.001
Mental fatigue	9.66 ± 2.62	12.17 ± 3.13	14.07 ± 3.78	<0.001
Reduced activities	8.46 ± 3.33	9.12 ± 2.54	9.22 ± 3.23	0.131
Motivation	8.94 ± 3.14	10.87 ± 2.76	11.11 ± 3.64	<0.001
MFI-K total score	41.36 ± 10.52	52.85 ± 9.63	61.23 ± 10.54	<0.001
VAS score	16.85 ± 18.19	33.02 ± 26.53	55.74 ± 32.28	<0.001
FSS score	20.59 ± 9.09	31.26 ± 10.25	43.81 ± 10.25	<0.001

## Data Availability

The data used to support the findings of this study are available from the corresponding author upon request.
